# Opposing Somatic and Dendritic Expression of Stimulus-Selective Response Plasticity in Mouse Primary Visual Cortex

**DOI:** 10.3389/fncel.2019.00555

**Published:** 2020-01-14

**Authors:** Taekeun Kim, Francesca A. Chaloner, Sam F. Cooke, Mark T. Harnett, Mark F. Bear

**Affiliations:** ^1^Department of Brain and Cognitive Sciences, Picower Institute for Learning and Memory, Massachusetts Institute of Technology, Cambridge, MA, United States; ^2^MRC Centre for Neurodevelopmental Disorders (CNDD), King’s College London, London, United Kingdom; ^3^Department of Basic and Clinical Neuroscience, Maurice Wohl Institute for Clinical Neuroscience, Institute of Psychiatry, Psychology and Neuroscience, King’s College London, London, United Kingdom; ^4^Department of Brain and Cognitive Sciences, McGovern Institute for Brain Research, Massachusetts Institute of Technology, Cambridge, MA, United States

**Keywords:** memory, habituation, primary visual cortex, mouse, novelty

## Abstract

Daily exposure of awake mice to a phase-reversing visual grating stimulus leads to enhancement of the visual-evoked potential (VEP) in layer 4 of the primary visual cortex (V1). This stimulus-selective response potentiation (SRP) resembles and shares mechanistic requirements with canonical long-term synaptic potentiation (LTP). However, it remains to be determined how this augmentation of a population response translates into altered neuronal activity of individual V1 neurons. To address this question, we performed longitudinal calcium imaging of layer 4 excitatory neurons in V1 and tracked changes associated with the induction and expression of SRP. We found no evidence for a net change in the fraction of visually responsive neurons as the stimulus became familiar. However, endoscopic calcium imaging of layer 4 principal neurons revealed that somatic calcium transients in response to phase-reversals of the familiar visual stimulus are reduced and undergo strong within-session adaptation. Conversely, neuropil calcium responses and VEPs are enhanced during familiar stimulus viewing, and the VEPs show reduced within-session adaptation. Consistent with the exquisite selectivity of SRP, the plasticity of cellular responses to phase-reversing gratings did not translate into altered orientation selectivity to drifting gratings. Our findings suggest a model in which augmentation of fast, short-latency synaptic (dendritic) responses, manifested as enhanced layer 4 VEPs, recruits inhibition to suppress cellular activity. Reduced cellular activity to the familiar stimulus may account for the behavioral correlate of SRP, orientation-selective long-term habituation.

## Introduction

Understanding how the cerebral cortex is changed by experience to influence the behavior of an organism is one of the dominant questions in neuroscience. Previous studies have shown that mouse primary visual cortex (V1) expresses experience-dependent plasticity that serves as a form of recognition memory that alters behavior. Mice will innately orient to and approach a computer monitor displaying novel, phase-reversing, high-contrast visual grating stimuli of a single orientation (Cooke et al., 2015). This behavioral response habituates when tested on successive days but returns if the grating is rotated to a novel orientation. This orientation-selective habituation (OSH) comprises a behavioral report of the formation of long-term visual recognition memory for the familiar stimulus. Phase-reversing stimuli that elicit OSH also trigger visual evoked potentials (VEPs) that can be monitored chronically over days *via* electrodes implanted in layer 4 of V1 (Frenkel and Bear, 2008). As OSH develops, the VEP in response to the familiar stimulus orientation grows, a phenomenon called stimulus-selective response potentiation (SRP; Frenkel et al., 2006). Experimental manipulations of V1 that disrupt SRP do the same to OSH. For example, both SRP and OSH require activation of N-methyl-D-aspartate receptors (NMDARs) in V1. Further, both are disrupted by local infusion of the Z-pseudosubstrate inhibitory peptide (ZIP; Cooke and Bear, 2010) and by manipulations of parvalbumin-containing (PV+) inhibitory interneurons (Kaplan et al., 2016). Thus, understanding how SRP is induced and expressed in V1 is likely to provide critical insight into visual recognition memory manifested as long-term behavioral habituation to familiar stimuli.

Much of what is known about SRP has come from the study of VEPs recorded in layer 4 of the visual cortex. VEPs are useful because they can be recorded through chronically implanted electrodes, enabling measures of absolute changes in response magnitude across days, and the relative simplicity of the method improves yield for mechanistic studies. Furthermore, the peak negativity of the VEP has been shown through current-source density analysis to reflect a synaptic current sink in layer 4 (Mitzdorf, 1985; Cooke et al., 2015). However, it is now understood that there is more to SRP than plasticity at feed-forward synapses (Cooke and Bear, 2014). For example, expression of SRP is mimicked and occluded by reducing activity in PV+ neurons, and by treatment with ketamine (Kaplan et al., 2016). To gain insight into how the cortical network is modified by visual experience, it is necessary to monitor the responses of individual neurons across days.

In the current study, we used *in vivo* calcium imaging to monitor activity at a cellular resolution across days as SRP was induced. This approach yielded a new view of how mouse V1 is modified by experience. Instead of response potentiation, we observed a robust depression and altered dynamics of cellular responses across days as the oriented stimulus became familiar. Like SRP and OSH, the altered response magnitudes and dynamics recovered when tested using a novel stimulus orientation. We also found that the modification of responses to familiar phase-reversing grating stimuli did not translate into altered orientation selectivity when assayed using drifting gratings, underscoring the exquisite stimulus selectivity of this plasticity. The findings that rapid dendritic responses recorded with VEPs are augmented while the slower somatic responses revealed by calcium imaging are depressed suggests a model in which inhibition in V1 is strongly recruited by visual stimuli recognized as familiar.

## Materials and Methods

### Animals

All experiments were performed in accordance with the guidelines of the National Institutes of Health and protocols approved by the Committee on Animal Care at the Massachusetts Institute of Technology. Mice were housed with food and water *ad libitum* and maintained on a 12 h light/dark cycle. EMX1.Cre mice (B6.129S2-*Emx1*^tm1(cre)Krj^/J, RRID:IMSR_JAX:005628) and GCaMP6f mice (*Igs7*^tm93.1(tetO-GCaMP6f)Hze^ Tg(Camk2a-tTA)1Mmay/J, RRID:IMSR_JAX:024108) were obtained from The Jackson Laboratory (Bar Harbor, ME, USA) and crossbred as needed in the animal facility at MIT. Only EMX1.GCaMP6f.tTA triple transgenic mice were used as the transgenic group and EMX1.GCaMP6 mice were used as wild-type littermate control animals. All EMX1.GCaMP6 mice were fed with a sterile doxycycline food pellet (200 mg/kg, Bio-Serv, Flemington, NJ, USA) until weaned and changed to a normal diet. Scnn1a.Cre mice (B6; C3-Tg(Scnn1a-cre)3Aibs/J, RRID:IMSR_JAX:009613) were used for endoscopic calcium imaging and were P28–35 at the time of first surgery to express GCaMP6. Both male and female mice were used for all experiments.

### Immunohistochemistry

Mice were deeply anesthetized with fatal plus (pentobarbital) and transcardially perfused with saline followed by 4% paraformaldehyde in 0.1 M phosphate-buffered saline. The brain was collected and post-fixed for 24 h in 4% paraformaldehyde in a cold room and then immersed for ~3 days in a 30% high sucrose solution. The brain was then sectioned into 50 μm coronal or sagittal slices using a vibratome (VT-1000S, Leica Biosystems, Richmond, IL, USA). Slices were incubated with a blocking solution (5% fetal bovine serum in 0.1 M PBS with 0.3% Triton X-100) on a shaker for 1 h at room temperature. After washing in PBS three times, sections were incubated with mouse anti-Parvalbumin primary antibody (P3088, Sigma–Aldrich, St. Louis, MO, USA, 1:1,000) at 4°C on a shaker overnight. Slices were then washed three times with PBS and incubated with fluorescent secondary antibodies (Alexa-568 conjugated goat anti-mouse IgG, A11004, Thermo Fisher Scientific, Waltham, MA, USA, 1:500; Hoechst 33,342, Life Technologies, New York, NY, USA, 1:2,000) on a shaker for 3 h at room temperature. Slices were washed three times with PBS and mounted on slides and coverslipped. Images were taken using a confocal fluorescence microscope (Olympus, Japan).

### *In vivo* Electrophysiology

Adult mice [postnatal day (P) 60–85] were anesthetized with vaporized isoflurane, 5% for induction and 1.5%–2% for maintenance. The hair overlying the scalp was shaved using hair clippers and eye ointment (Puralube vet ointment, Dechra, Leawood, KS, USA) was applied to prevent the animal’s eyes from drying out. The animal was positioned into a stereotaxic frame and warmed with a heating pad. The scalp was sterilized with betadine and 70% alcohol swabs (BD alcohol swabs, BD). Prior to surgical incision, 1% lidocaine hydrochloride anesthetic was injected under the scalp. A steelhead post was affixed to the anterior of the skull using cyanoacrylate glue. Burr holes (<0.5 mm) were drilled over binocular V1 (2.7–3.2 mm lateral of lambda). Tapered tungsten recording microelectrodes (FHC, ME) were then implanted in both hemispheres, 450 μm below the cortical surface. A silver wire (A-M Systems Inc., Sequim, WA, USA) reference electrode was positioned on the dura overlying prefrontal cortex. The head post and electrodes were secured with cyanoacrylate glue and dental cement. Mice were allowed to recover for at least 48 h prior to habituation to restraint in the recording apparatus. During habituation sessions on two consecutive days, mice viewed a gray screen for 30 min/day. VEP recordings in response to phase-reversing high-contrast visual grating stimuli (0.05 cyc/degree) were amplified and digitized using the Recorder-64 systems (Plexon Inc., Dallas, TX, USA). Two channels were dedicated to recording EEG/VEPs from V1 of each implanted hemisphere at 1 kHz sampling frequency with a 500 Hz lowpass filter. Data were extracted from the binary storage files and analyzed using customized Matlab software. VEPs were averaged across all phase reversals within a block and the trough-peak difference during the 200-ms period following phase reversal was measured.

### *In vivo* Two-Photon Calcium Imaging

Young adult GCaMP6f transgenic mice (P30–35) were anesthetized and prepared as described above up to the surgical incision. Following incision, a lidocaine (2%) and epinephrine (1:50,000) solution was applied onto the periosteum and the exposed area of skull gently scraped with a scalpel blade. Then, a 3 mm craniotomy was made over binocular V1 and a sterile 3 mm round glass coverslip (CS-3R-0, Warner Instruments, Hamden, CT, USA) was gently laid on top of the exposed dura mater. The coverslip was secured with cyanoacrylate glue and a stainless steel head post was attached to the skull. Once the glue had set, dental acrylic (C&B Metabond Quick Adhesive Cement System, Parkell, NY, USA) was mixed and applied throughout the exposed skull surface.

Two to three weeks following craniotomy surgery, the mice were habituated to the behavior restraint apparatus in front of a gray screen with the objective lens of the two-photon microscope positioned on the head plate for 30 min for two consecutive days. A Ti:sapphire laser (Coherent, Santa Clara, CA, USA) was used for imaging at a wavelength of 920 nm. Photomultiplier tubes (Hamamatsu, Japan) and the objective lens (20×, 0.95 NA, XLUMPLFLN, Olympus, Japan) were used to detect fluorescence images. Calcium image recordings were triggered by time-locked TTL pulses generated from USB-1208fs (Measurement Computing, Norton, MA, USA) using the Prairie view and TriggerSync Software (Bruker, CA, USA) and imaged at a frequency of 4 Hz at the depth of ~350 μm in V1. The size of the imaging field of view was ~300 × 300 μm^2^ at 256 × 256 pixels.

Acquired time series of calcium imaging files were processed using ImageJ and customized MATLAB software. All recorded files were concatenated and registered using TurboReg plugin (Fiji ImageJ) to stabilize motion movement and regions of interest (ROIs) were selected manually by going through all recorded frames using Time Series Analyzer plugin (Fiji ImageJ). Baseline calcium responses, F_0_, were calculated as the mean value of a minimal 5-s bin acquired during gray screen for each ROI and ΔF/F_0_ were calculated accordingly. Unless otherwise indicated, background ΔF/F_0_ at each time point was then calculated as the mean of 15 ROIs selected from the background and subtracted from all the time series of ΔF/F_0_ of cellular ROIs. Calcium transients were ΔF/F_0_ of cellular ROIs which were higher than three times the standard deviation (SD) of the background ΔF/F_0_.

### Viral Infection and Prism Implantation

Juvenile Scnn1a.Cre mice (P28–35) were anesthetized and prepared as described above up to surgical incision. Burr holes (<0.5 mm in diameter) were drilled in the skull over binocular V1 and Adeno-associated virus containing the GCaMP6f gene (pAAV1.Syn.Flex.GCaMP6f.WPRE.SV40, 100833-AAV1, Addgene, Watertown, MA, USA) was loaded into a glass micropipette with a tip diameter of 40–50 μm attached to a Nanoject II injection system (Drummond Scientific, Broomall, PA, USA). The micropipette was then inserted into binocular V1 layer 4 at depths of 350, 400, 450 μm below pial surface and ~50 nl virus was delivered at each depth. After ~3 weeks of recovery to allow for gene expression, mice were anesthetized and a steelhead post was affixed to the anterior skull using cyanoacrylate glue. A 1.5 mm in diameter craniotomy was made over the virus injection area. Dura was removed and a 1 mm incision was made horizontally using a microblade and a microprism lens (ProView Prism probe, ID: 1050-002204, Inscopix, Palo Alto, CA, USA; Murayama et al., 2007; Andermann et al., 2013) was then embedded along the incision. Dental acrylic was mixed with black ink and applied throughout the skull surface. After 5–6 days, the baseplate (ID:1050-002192, Inscopix, Palo Alto, CA, USA) of a miniaturized integrated fluorescent microscope (nVista, Inscopix, Palo Alto, CA, USA; Ziv et al., 2013) was mounted and secured onto the embedded prism probe under visual guidance using a temporarily attached microscope in order to determine the optimal fluorescent field of view.

### *In vivo* Endoscopic Calcium Imaging

Three to four days following the baseplate attachment, mice were habituated to the behavior restraint apparatus in front of a gray screen for 30 min for two consecutive days. Calcium images were obtained at a frequency of 30 Hz using nVista HD software (Inscopix, Palo Alto, CA, USA). Five volt TTL pulses were generated for the initial 1.15 s of each 2-s visual stimulus through USB-1208fs. Images were collected during the 1.15 sTTL pulse in order to time-lock acquisition with the visual stimulus and to prevent possible bleaching caused by the LED light. The size of the imaging field of view was approximately 900 × 650 μm^2^ and the focal plane was determined by adjusting the microscope turret until the largest population of clearly fluorescent cells were observed.

Acquired time series of calcium images were processed using Inscopix Data Processing Software (Inscopix, Palo Alto, CA, USA), ImageJ, and customized MATLAB. All fragmentized recorded files to each phase reversal of the visual stimulus were concatenated and spatially down-sampled by 2-fold along spatial dimensions to reduce file size and noise and then a spatial band-pass filter was applied (0.005–0.5/px). Processed files were extracted as tiff files and went through analysis in the same manner as two-photon calcium imaging data using customized MATLAB. Baseline calcium responses, F_0_, were calculated as the mean value of a minimal 1-s bin acquired during gray screen for each ROI. The rest of the process for calculating ΔF/F_0_ was the same as described in *in vivo* two-photon calcium imaging methods section. For orientation tuning (OT) analyses, mean ΔF/F_0_ of individual ROI responses (R) were calculated based on the magnitude of calcium responses during six, 3-s sweeps of a drifting grating at each of eight different stimulus directions interleaved by 5-s of a gray screen. Mean response to each corresponding direction were fit with bimodal Gaussian function (Carandini and Ferster, 2000),

R(θ)=Rbase+Rprefe−((θ−θpref)22σ2)   +Rorthoe−((θ−θpref+180)22σ2)

where θ is the stimulus orientation expressed as angular values between 0° and 360°; *R*_base_, baseline; *R*_pref_ and *R*_ortho_ are the response amplitudes at θ_pref_ and θ_pref+180_; σ, the tuning width. The tuning width for the preferred orientation is calculated as the full width at half maximum (FWHM) of the Gaussian function, $2\sqrt{2\ln2\sigma }$. Orientation selectivity index (OSI) was calculated as (*R*_pref_ − *R*_ortho_)/(*R*_pref_ + *R*_ortho_).

### Neuropil Analysis

The 15 ROI per animal that had been selected for background measurement in the somatic endoscopic time series were analyzed separately to estimate neuropil responses to phase-reversing stimuli. Baseline calcium responses of neuropil, F_0_, were calculated as the mean value of a minimal 1-s bin acquired during the gray screen, and ΔF/F_0_ of neuropil was calculated at each time point.

### Visual Stimuli

Visual stimuli were generated and presented on a computer monitor using software custom-written in either C++ for interaction with a VSG2/2 card (Cambridge Research Systems, Rochester, UK) or Matlab (MathWorks, Natick, MA, USA) using the PsychToolbox extension in order to control stimulus drawing and timing. The display was positioned 20 cm in front of the head-fixed mouse, and the visual field size was 92° × 66°, with a mean luminance of 27 cd/m^2^. Visual stimuli consisted of full-field, 100% contrast, sinusoidal gratings phase reversing at a frequency of 0.5 Hz. For the large cohort of mice used to assess VEP adaptation across phase reversals, stimuli reversed at 2 Hz. Grating stimuli spanned the full range of monitor display values between black and white, with gamma-correction to ensure constant total luminance during both the gray and patterned stimulus screen. Throughout, stimulus orientation varied such that a novel orientation was always a minimum of 60° different from any previously experienced by a given mouse. For most experiments described, each stimulus block consisted of 60 phase reversals followed by a 30 s interleaved gray screen with the exception for when orientation tuning was measured. For orientation tuning measurements, full-field, 100% contrast, drifting sinusoidal gratings at 2 Hz with eight different directions were presented within each block. Each direction (0°, 45°, 90°, 135°, 180°, 225°, 270°, 315°) of the drifting grating was presented for 3 s and interleaved by 5 s of gray screen. Eight directions were presented pseudo-randomly within a block and repeated six times.

### Experimental Design and Statistical Analysis

**Figure 1:**
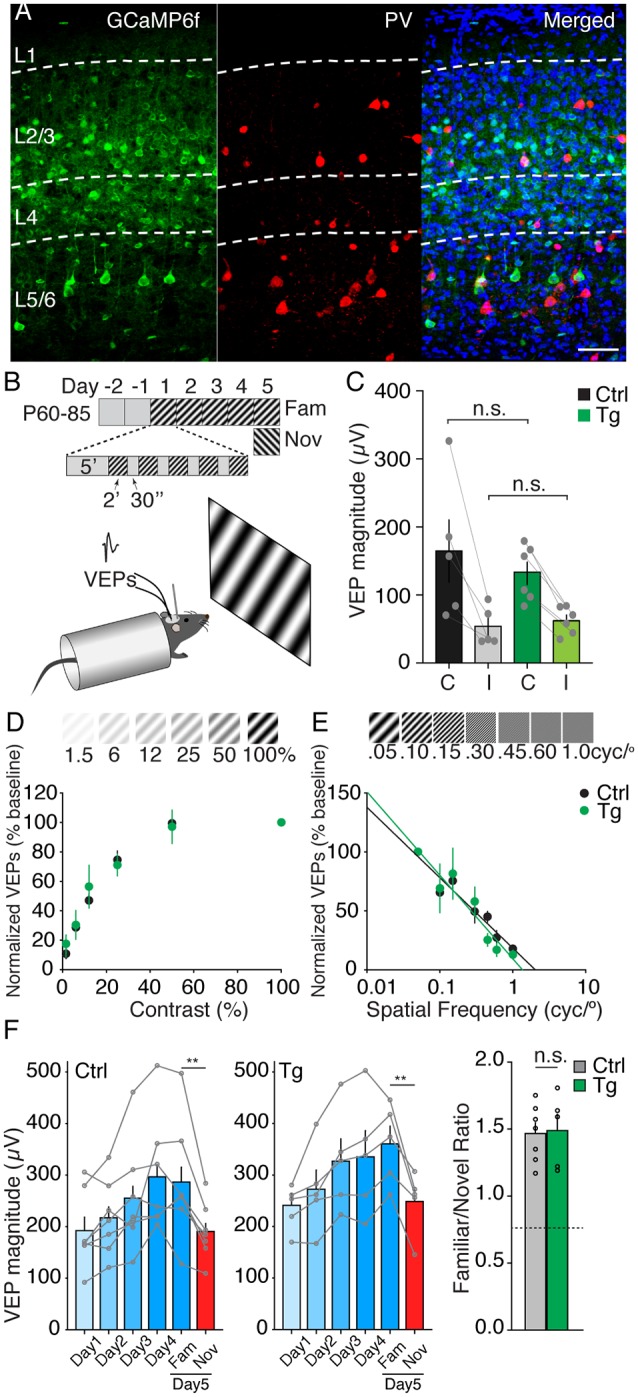
GCaMP6f transgenic mice show normal visual properties and plasticity in binocular V1. **(A)** Confocal microscopic images of binocular V1 in the GCaMP6f transgenic mouse demonstrating transgene expression confined to Emx1-expressing principal neurons. Principal cells expressing GCaMP6f are green, parvalbumin-expressing interneurons are red, and Hoechst-stained neurons are blue. Scale bar, 50 μm. **(B)** Timeline of experimental paradigm and recording schematic. **(C)** Visual-evoked potential (VEP) magnitudes to contralateral (C) and ipsilateral (I) eye stimulation are comparable in wild-type control (black, *n* = 5) and transgenic mice (green, *n* = 6). (Interaction between genotype and eye, *F*_(1,9)_ = 1.517, *p* = 0.249; Comparison between eyes, *F*_(1,9)_ = 27.92, *p* = 0.0005; Comparison between genotypes, *F*_(1,9)_ = 0.162, *p* = 0.696 two-way ANOVA). **(D,E)** Contrast sensitivity and visual acuity tests of transgenic (green, *n* = 6) and wild-type control mice (black, *n* = 6) demonstrate comparable visual function between genotypes. Contrast and acuity measure are normalized to a 100% contrast and 0.05 cyc/° stimulus, respectively. **(F)** Selective response potentiation (SRP) expression is comparable between wild type and transgenic mice. (Comparison between response to familiar and novel stimulus on day 5, control mice, *n* = 7, transgenic mice, *n* = 5, Interaction between genotype and stimulus type, *F*_(1,10)_ = 0.213, *p* = 0.655; Comparison VEPs to familiar and novel visual stimulus, *F*_(1,10)_ = 35.23, *p* = 0.0001; Comparison between WT and Tg, *F*_(1,10)_ = 2.12, *p* = 0.176 two-way ANOVA; Familiar/novel response ratio, unpaired *t*-test n.s. *p* = 0.887). Error bars indicate SEM. *p < 0.05, **p < 0.01, ***p < 0.001, n.s. = *p* > 0.05.

All data are expressed as the mean ± SEM and *n* represent individual animals unless mentioned otherwise. Matlab, SigmaPlot and Prism were used for parametric statistical analysis. Student’s two-tailed paired *t*-tests were calculated for comparisons between two groups. Two-way ANOVA was used for data presented in Figures 1C,F. Also, for multiple comparisons data presented in Figures 4, 5, one-way ANOVA followed by Tukey’s *post hoc* tests with Bonferroni correction were used. *P* < 0.05 was used as the threshold for statistical significance for all parametric comparisons and exact values are presented within the results section.

**Figure 2:**
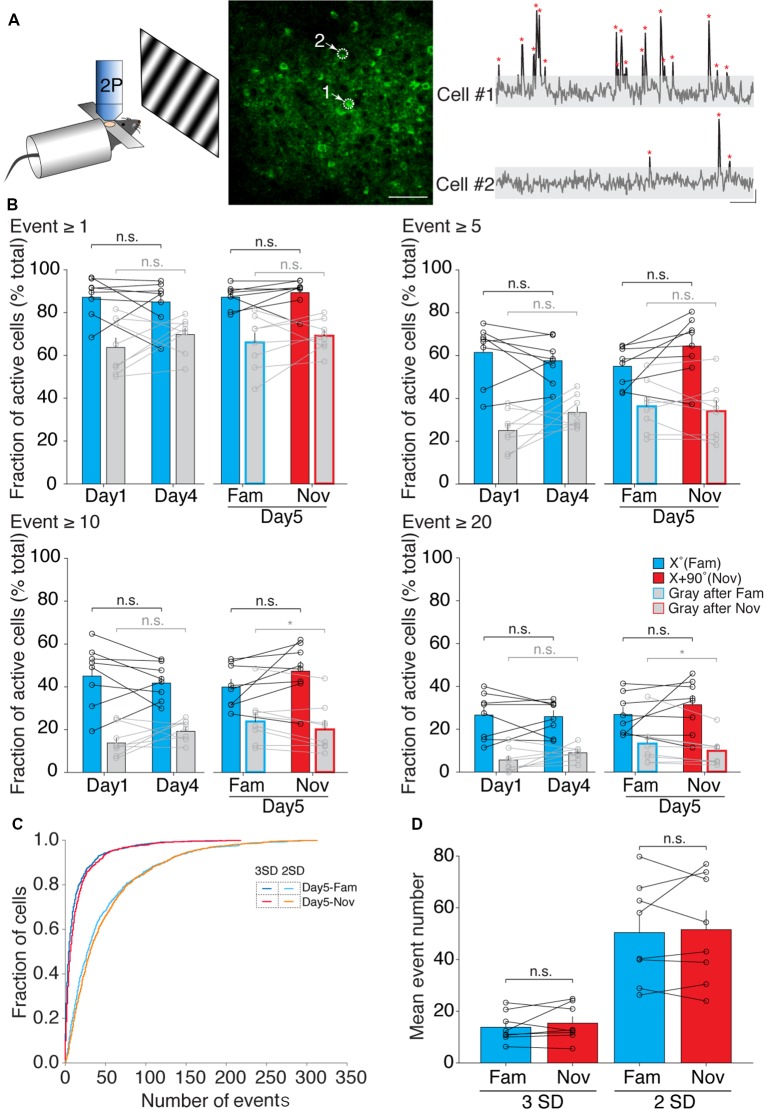
The fraction of active neurons in layer 4 of binocular V1 during a familiar visual stimulus presentation does not change following SRP induction. **(A)** Left: schematic of *in vivo* two-photon calcium imaging setup with visual stimulation. Middle: two-photon image of a field of view. White arrows and dotted circles indicate two individual cells undergoing imaging. Scale bar, 50 μm. Right: example of calcium traces from the two cells indicated in the middle image. Calcium transient peaks (red asterisk), 3 standard deviations (SDs) above the noise (gray shading), were used for analysis. Scale bar, 10 s, 20% ΔF/F. **(B)** The fraction of active neurons on day 1 and day 4 following SRP induction, as well as on day 5 during presentation of the familiar (Fam) and novel (Nov) visual stimulus does not change irrespective of whether neurons show few (≥1) or many (≥20) calcium events during presentation of the grating visual stimulus (Paired *t*-test, n.s.). Gray bars indicate the fraction of responsive neurons during the time when a gray screen was present on the monitor. **(C)** Cumulative distribution of the number of calcium transients of all cells (*n* = 929 cells from eight animals) on day 5 during the familiar visual stimulus and novel, with two different thresholds (3 SD: blue and red; 2 SD: cyan and orange) for calcium event. **(D)** Mean event number of all cells during presentation of the familiar visual stimulus and novel stimulus on day 5, with two different thresholds (3 SD and 2 SD) for calcium event. Averaged by animal (*n* = 8; 3 SD: familiar, 13.79 ± 2.03; novel, 15.35 ± 2.47, paired *t*-test *p* = 0.384; 2 SD: familiar, 50.41 ± 6.85; novel, 51.54 ± 7.28, paired *t*-test *p* = 0.736). Error bars indicate SEM. *p < 0.05, n.s. = *p* > 0.05.

**Figure 3:**
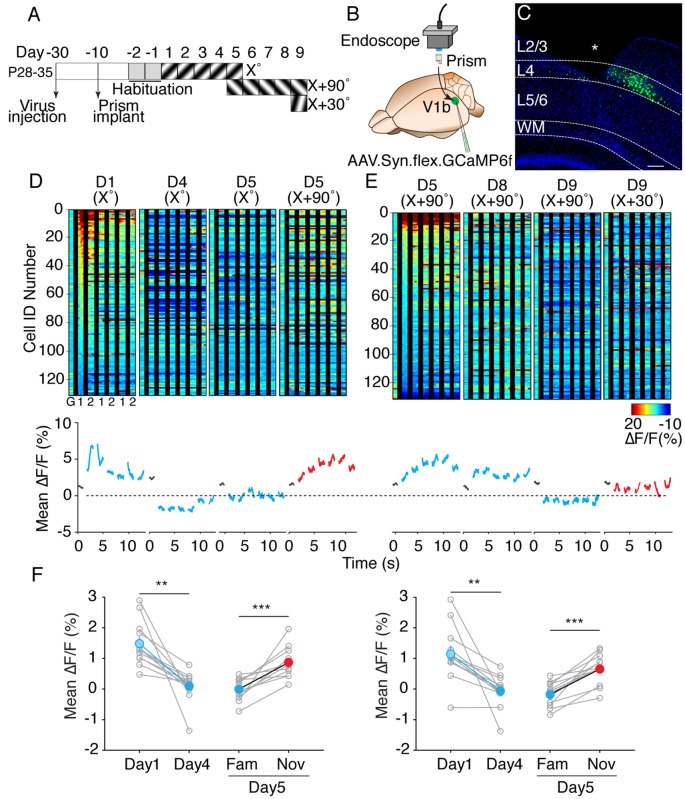
V1 layer 4 principal neurons show a decrease in calcium response magnitude to the familiar visual stimulus following SRP induction. **(A)** Timeline of experimental procedure and training paradigm. **(B)** Schematic of prism implantation in V1 and endoscopic imaging. **(C)** Confocal image of prism implanted Scnn1a mouse with GCaMP6f virus injection targeted to layer 4 principal neurons. Asterisk indicates a tract of prism. Scale bar, 100 μm. **(D)** Top: representative heat map of cellular calcium responses from all neurons from one animal across training days (D) to the presented visual stimulus orientation (°) as depicted in panel **(A)**. G indicates gray screen, 1 indicates one phase-reversal of the visual stimulus and 2 indicates subsequent phase-reversal. Bottom: mean magnitude of calcium responses at each time point from the panel above. Gray traces indicate the presence of a gray screen. **(E)** Same analyses as performed in Figure 3D. Cell IDs are reordered in descending order of activity in response to X + 90° on day 5. **(F)** Left panel: mean magnitude of calcium responses of all imaged neurons (*n* = 1,842) from all animals (*n* = 12) during the 50–1,200 ms period following onset and subsequent phase reversals of the visual stimulus. There is an overall decrease in the mean magnitude of calcium response between day 1 and day 4 (Paired *t*-test, *p* = 0.001). Mean Ca^2+^ response on day 5 to the familiar visual stimulus is also significantly reduced compared to the mean response to the novel visual stimulus (Paired *t*-test, *p* = 0.0003). Right panel: mean magnitude of Ca^2+^ responses of all recorded neurons from all animals during the 50–150 ms period following onset and subsequent phase reversals of the visual stimulus. Mean magnitude comparison between day 1 and day 4 is significant (Paired *t*-test, *p* = 0.003). Day 5 familiar and novel comparison is also significant (Paired *t*-test, *p* = 0.00004). Error bars indicate SEM. **p < 0.01, ***p < 0.001.

**Figure 4:**
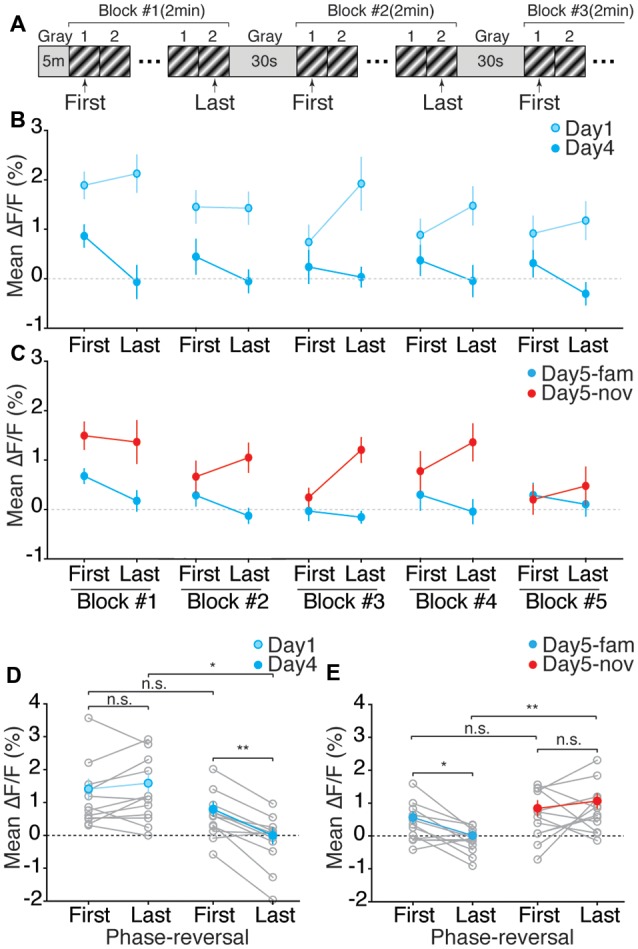
The cellular Ca^2+^ response of V1 layer 4 neurons is reduced both within blocks and across days of familiar visual stimulus presentation during induction of SRP. **(A)** Schematic of daily visual stimulus presentation and indication of the first and the last phase-reversal within a stimulus block used for analysis. Gray indicates a gray screen, 1 indicates one phase-reversal of the visual stimulus, 2 indicates subsequent phase-reversal. **(B)** Mean amplitude of Ca^2+^ responses from 12 animals for the first and last phase-reversal of the visual stimulus in each block of five blocks of visual stimulus presentation on day 1 and day 4 of training, and **(C)** during the presentation of the familiar and novel visual stimulus on day 5 of training. **(D)** Mean amplitude of Ca^2+^ responses (*n* = 1,842 cells) from all animals (*n* = 12) for the last phase-reversal of the visual stimulus on day 4 is reduced compared to the responses to the first phase-reversal on the same day and is also reduced as compared to Ca^2+^ responses for the last phase-reversal on day 1 (One-way ANOVA with Tukey’s *post hoc* test: last phase-reversal, day 1–day 4, *p* = 0.016; first and last phase-reversal on day 4, *p* = 0.002). **(E)** Mean amplitude of Ca^2+^ responses of all animals to the last phase-reversal of the familiar visual stimulus (blue circle) on day 5 is significantly reduced compared to the response to the first phase-reversal of the familiar visual stimulus and also compared to the response to the novel visual stimulus (red circle; One-way ANOVA, Tukey’s *post hoc* test: last phase-reversal, day 5 fam—nov, *p* = 0.002; first and last phase-reversal of fam, *p* = 0.039). Error bars indicate SEM. *p < 0.05, **p < 0.01, ***p < 0.001, n.s. = *p* > 0.05.

**Figure 5:**
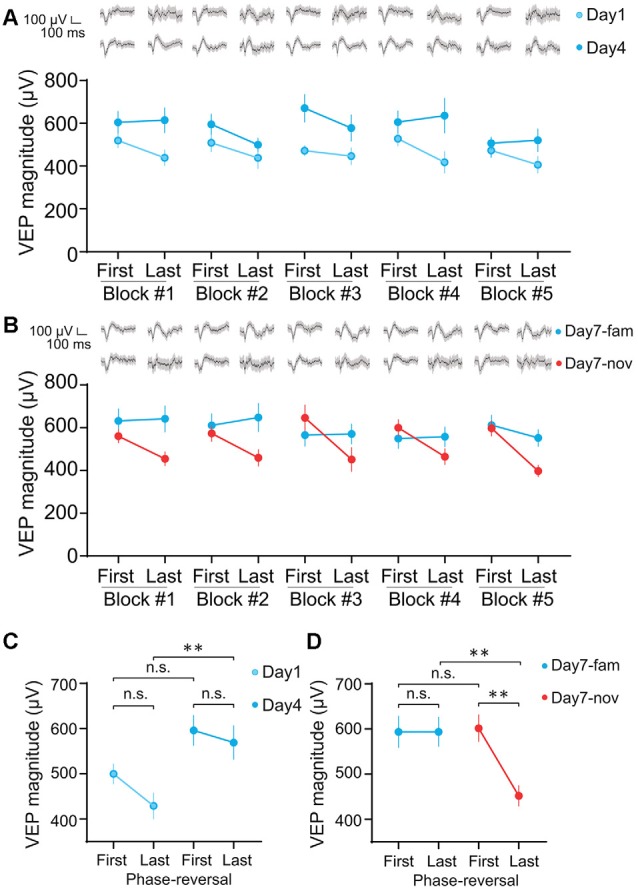
Layer 4 VEPs recorded during SRP induction and expression exhibit short-term adaptation during novel stimulus presentation. **(A)** Mean magnitude of VEP responses from 34 animals for the first and last phase-reversal of the visual stimulus in each of five blocks of visual stimulus presentation on day 1 (open circles) and day 4 (closed circles) of training, and **(B)** during presentation of the familiar (blue circles) and novel visual stimulus (red circles) on final test day 7. Averaged VEPs are shown at the top of each panel. **(C)** Mean magnitude of VEP responses from 34 animals for the last phase-reversal of the visual stimulus on day 1 is reduced compared to the responses to the first phase-reversal on the same day (open circles) but does not show similar adaptation on day 4 (closed circles; 1-way ANOVA with Tukey’s *post hoc* test for multiple comparisons: Day 1 first and last, *p* = 0.366; Day 4 first and last, *p* = 0.926; First on day 1 and day 4, *p* = 0.124; Last on day 1 and day 4, *p* = 0.008). **(D)** On the final test day 7 after SRP, the mean magnitude of VEP responses from 34 animals for the last phase-reversal of a novel oriented stimulus is reduced compared to VEPs elicited by the first phase-reversal of the same stimulus (red circles). This adaptation effect is not observed for the highly familiar orientation on the same day (blue circles; 1-way ANOVA with Tukey’s *post hoc* test for multiple comparisons: familiar first and last, *p* = 1.00; Novel first and last, *p* < 0.003; First for familiar and novel, *p* = 0.997; Last for familiar and novel, *p* = 0.006). Error bars indicate SEM. *p < 0.05, **p < 0.01, n.s. = *p* > 0.05.

## Results

### Postnatal Conditional Expression of GCaMP6f Does Not Affect Visual Properties or Experience-Dependent Plasticity in V1

The objective of our study was to assess how the activity of layer 4 neurons in V1 is changed over days as an initially novel stimulus becomes familiar. Our first approach entailed monitoring cellular activity in cortical neurons across days using two-photon imaging of the calcium indicator GCaMP6f expressed in transgenic mice (see “Materials and Methods” section). The breeding strategy was designed to confine GCaMP6f gene expression to Emx1-expressing excitatory principal cells in V1 (Figure 1A). We also used the doxycycline (dox) dependent tet-off expression system to delay the expression of the GCaMP6f gene in order to prevent any possible interference with the early development of V1. Following the withdrawal of Dox from the diet at postnatal day (P) 21, awake, head-fixed animals were subjected to a battery of electrophysiological tests to assess the integrity of the visual system (Figure 1B). Mouse binocular V1 normally has a contralateral eye bias wherein VEPs elicited by visual stimulation of the contralateral eye are ~3-fold larger than ipsilateral eye responses (Porciatti et al., 1999). Our measurements of VEPs in layer 4 confirmed that the contralateral eye bias and VEP magnitudes to visual stimulation of each eye were comparable in transgenic and wild-type control littermate mice (Figure 1C, contralateral, control, 164.8 ± 45.9 μV, Tg, 132.0 ± 16.6 μV; ipsilateral, control, 53.1 ± 12.7 μV, Tg, 62.6 ± 8.3 μV; no genotype by eye interaction, *F*_(1,9)_ = 1.517, *p* = 0.249; significant main effect of eye, *F*_(1,9)_ = 27.92, *p* = 0.0005; no main effect of genotype, *F*_(1,9)_ = 0.162, *p* = 0.696 two-way ANOVA). Presentation of phase-reversing grating stimuli at 6 different contrasts at 0.05 cyc/° (1.5%, 6%, 12%, 25%, 50%, 100%), revealed comparable contrast sensitivity of transgenic mice compared to wild-type littermates (Figure 1D; Table 1). Visual acuity, tested by presenting 100% contrast stimuli at 7 different spatial frequencies (0.05 cyc/°, 0.1 cyc/°, 0.15 cyc/°, 0.3 cyc/°, 0.45 cyc/°, 0.6 cyc/°, 1.0 cyc/°), was also comparable between transgenic and wild-type littermate control animals (Figure 1E; Table 2).

**Table 1:** Summary of contrast sensitivity.

	Stimulus contrast (%)					
Genotype	1.5	6.0	12	25	50	100
	Normalized VEP magnitude (%)					
Ctrl	10.8 ± 3.2	28.6 ± 4.2	47.0 ± 4.1	74.6 ± 6.0	99.4 ± 2.6	100
Tg	17.5 ± 5.9	30.4 ± 9.7	56.4 ± 14.5	71.0 ± 7.4	97.0 ± 11.3	100
*p*-value	0.367	0.872	0.556	0.717	0.846	N/A

**Table 2:** Summary of visual acuity.

	Stimulus spatial frequency (cycle/°)						
Genotype	0.05	0.10	0.15	0.30	0.45	0.60	1.00
	Normalized VEP magnitude (%)						
Ctrl	100	65.5 ± 6.2	75.4 ± 6.5	49.3 ± 9.7	45.0 ± 4.5	27.2 ± 6.0	17.8 ± 2.8
Tg	100	69.0 ± 20.7	81.4 ± 21.6	57.8 ± 12.1	25.2 ± 5.2	16.8 ± 5.4	12.7 ± 3.8
*p*-value	N/A	0.877	0.798	0.597	0.017	0.225	0.306

Although expression of GCaMP has been reported to leave synaptic plasticity intact (Huber et al., 2012), we considered the possibility that endogenous expression of a calcium buffer might interfere with SRP induction. Therefore we used standard electrophysiological measures to investigate the induction and expression of SRP in transgenic animals (Frenkel et al., 2006). Briefly, animals had microelectrodes implanted in layer 4 of binocular V1 and the magnitudes of VEPs elicited by phase-reversing grating visual stimulus of the same orientation across days were assessed (Figure 1B). Normally, VEPs progressively increase to the experienced (familiar) stimulus orientation following four consecutive days of exposure, and VEPs elicited by a novel oriented visual stimulus presented on day 5 are comparable in magnitude to the VEPs that were elicited by the familiar stimulus on day 1. In our experiments, both wild-type control and transgenic mice showed potentiation of the VEPs across days elicited by the familiar visual stimulus, and the ratio of their familiar/novel responses on day 5 of testing demonstrated that SRP expression was comparable between genotypes (Figure 1F, from day 1 to day 4, no genotype by time interaction, *F*_(3,30)_ = 0.407, *p* = 0.749; significant main effect of time, *F*_(3,30)_ = 15.96, *p* < 0.0001; no main effect of genotype, *F*_(1,10)_ = 1.118, *p* = 0.315 two-way ANOVA; day 5 familiar and novel, control : day 5 familiar, 286.6 ± 28.8 μV; day 5 novel, 190.5 ± 15.5 μV; Tg: day 5 familiar, 360.7 ± 34.3 μV; day 5 novel, 248.4 ± 27.3 μV; no genotype by stimulus type interaction, *F*_(1,10)_ = 0.213, *p* = 0.655; significant main effect of stimulus type, *F*_(1,10)_ = 35.23, *p* = 0.0001; no main effect of genotype, *F*_(1,10)_ = 2.12, *p* = 0.176 two-way ANOVA; familiar/novel ratio, control, 1.47 ± 0.08, Tg, 1.49 ± 0.12, *p* = 0.887 unpaired *t*-test). Combined, these findings demonstrate that GCaMP6f transgenic mice have visual response properties and long-term cortical plasticity comparable to their wild-type control littermates.

### The Fraction of Active Neurons During the Presentation of the Familiar Visual Stimulus Does Not Change Following SRP Induction

The observed growth of the VEP that occurs across days when an initially unexpected visual stimulus becomes familiar led us to hypothesize that more neurons might become active when this stimulus is on. To test this hypothesis, head-fixed mice were positioned in front of a computer monitor, and hundreds of GCaMP6f-expressing neurons were identified in layer 4 of V1. Then, the calcium responses, defined as events ≥3 SD above the baseline fluorescence (Chen et al., 2013), were tracked across days of SRP induction during the presentation of both the phase-reversing sinusoidal grating stimuli and interleaved gray screen (Figure 2A). We measured the number of active neurons during the presentation of the visual stimulus on each day of training, and applied various criteria to categorize a neuron as “active.” The least stringent requirement was that only one or more calcium transients occurred over the course of 10 min of visual stimulation. Additional criteria included requirements that ≥5, 10, or 20 calcium transients were observed in the neurons during the entire period of visual stimulation on a given day. Regardless of the criterion used, we found that there was no significant change in the fraction of active neurons when comparing day 1 and day 4 during the presentation of the trained visual stimulus. Similarly, the fraction of active neurons observed on day 5 during the presentation of the familiar and novel visual stimulus did not differ (Figure 2B; Table 3). The only difference observed was a slight increase in active neurons during gray screen epochs that followed the presentation of the familiar visual stimulus, using the activity criteria of ≥10 events (Table 3). This may reflect an increase in spontaneous neural activity following visual habituation, as previously reported (Miller et al., 2018). We also examined the possibility that we may have missed a difference because of the criterion used to accept a discrete event by reanalyzing the data using an event threshold of ≥2 SD of baseline noise (Figures 2C,D). Regardless of which analysis was applied, the results of these experiments do not support the hypothesis that SRP induction is accompanied by recruitment of more neurons during familiar visual stimulus viewing.

**Table 3:** Comparison summary of fraction of active neurons.

		Paired *t*-test (*p*-value)			
Screen	Comparison	Event ≥ 1	Event ≥ 5	Event ≥ 10	Event ≥ 20
Visual stimulus	Day1–Day4	0.62	0.35	0.45	0.81
	Day5 Fam-Nov	0.44	0.07	0.10	0.28
Gray	Day1–Day4	0.26	0.10	0.15	0.18
	Day5 Fam-Nov	0.45	0.44	0.02	0.03

### Evoked Somatic Calcium Responses Are Reduced as Visual Stimuli Become Familiar

Because of the limited scanning speed and sampling rate of our two-photon imaging system, the first experiment could only reveal changes in overall cellular activity present during the entire period of continuous visual stimulation. However, SRP is an enhancement in the magnitude of the VEP elicited 50–150 ms following each phase-reversal of the visual stimulus. Therefore, the question remained as to how the activity of neurons changes as a function of SRP induction when the analysis is performed on calcium events that are time-locked to each phase-reversal. To address this question, we used one-photon endoscopy combined with implantation of a prism probe gradient-index (GRIN) lens to reach layer 4 of V1 (Murayama et al., 2007; Andermann et al., 2013). GCaMP6f was expressed exclusively in the excitatory neurons of layer 4 by infecting V1 of P28–35 *Scnn1a-Cre* transgenic mice (Madisen et al., 2010) with Cre-dependent adeno-associated GCaMP6f virus (AAV.flex.GCaMP6f). After allowing ~3 weeks for optimal gene expression, the prism probe GRIN lens was implanted immediately adjacent to the virus injection site (Figures 3A,B). This strategy allowed us to image excitatory principal cells in layer 4 of V1 expressing the GCaMP6f gene (Figure 3C).

The design of these experiments is described in Figure 3A. Following habituation to restraint, the animals were given a standard SRP protocol, comprising daily exposure to phase-reversing gratings at a constant orientation (X° was either 45° or 135°) for four consecutive days. On day 5, responses to the familiar (X°) and a novel (X + 90°) visual stimulus were obtained. Thereafter, the X + 90° visual stimulus was presented for 4 additional days in order to induce SRP to this second stimulus orientation. On the ninth day of testing, responses to the now-familiar X + 90° stimulus were compared with those evoked by a new novel visual stimulus (X + 30°).

These experiments revealed that the calcium responses evoked by the X° gratings were reduced in layer 4 neurons over the course of 4 days as the stimulus became familiar (Figure 3D). This depression of the calcium response was not attributable to photo-bleaching, an overall reduction of cellular excitability, or simple regression to the mean, as responses to the novel X + 90° visual stimulus were significantly greater compared to the responses to the familiar visual stimulus on the 5th day of testing. This familiarity-induced reduction of calcium responses was also present following the second SRP induction protocol to the orientation X + 90° (Figure 3E).

The findings of reduced cellular responses with increasing stimulus familiarity were consistent across 12 mice (1,842 neurons total). In Figure 3F, the mean X° response amplitude across neurons for each mouse is plotted for day 1 and day 4, and for day 5 novel (X + 90°) vs. familiar (X°). Regardless of whether the mean was calculated over the entire observation period of 50–1,200 ms post phase-reversal (left panel), or was limited to events with a short latency of 50–150 ms (right panel), the data clearly showed that the cellular correlate of learned stimulus familiarity is a decreased calcium response (Figure 3F, left panel: day 1 ΔF/F = 1.48 ± 0.21%; day 4, 0.09 ± 0.15%; *p* = 0.001 paired *t*-test; day 5 familiar, −0.01 ± 0.10%; novel, 0.87 ± 0.14%; *p* = 0.0003 paired *t*-test; right panel: day 1ΔF/F = 1.14 ± 0.26%; day 4, −0.07 ± 0.15%; *p* = 0.003 paired *t*-test; day 5 familiar, −0.18 ± 0.11%; novel, 0.65 ± 0.14%; *p* = 0.00004 paired *t*-test).

### Calcium Responses to Repetitive Stimulation Differ for Familiar and Novel Stimulus Orientations

In addition to the decrease in mean response amplitude, we also observed different response dynamics during visual stimulation when the stimulus became familiar. A consistent observation across animals (*n* = 12) was that for familiar stimuli, the evoked calcium transients elicited by each phase reversal showed adaptation within each block of stimulation (comprising 60 phase reversals at 0.5 Hz). This within-block adaptation was not observed when the stimulus was novel (Figures 4A–C). Thus, the familiar-novel response amplitude differences were greatest for phase reversals at the end of a block (Figures 4D,E). This conclusion holds regardless of whether we compared responses to the same orientation on days 1 and 4 (Figure 4D, day 1 first ΔF/F = 1.41 ± 0.30%; day 1 last, 1.59 ± 0.30; day 4 first, 0.79 ± 0.15%; day 4 last, −0.003 ± 0.25%; 1-way ANOVA with Tukey’s *post hoc* test for multiple comparisons: day 1 first and last, *p* = 0.747; day 4 first and last, *p* = 0.002; first on day 1 and day 4, *p* = 0.518; last on day 1 and day 4, *p* = 0.016) or compared familiar and novel orientations on day 5 (Figure 4E, day 5 familiar first ΔF/F = 0.56 ± 0.18%; day 5 familiar last, 0.01 ± 0.12; day 5 novel first, 0.84 ± 0.23%; day 5 novel last, 1.06 ± 0.23%; 1-way ANOVA with Tukey’s *post hoc* test for multiple comparisons: day 5—familiar first and last, *p* = 0.039; day 5—novel first and last, *p* = 0.859; first on day 5—familiar and novel, *p* = 0.779; last on day 5—familiar and novel, *p* = 0.002).

### VEP Responses to Repetitive Stimulation Vary With Familiarity

Although averaged VEP recordings are typically used to measure SRP (Frenkel et al., 2006; Cooke and Bear, 2010; Aton et al., 2014; Cooke et al., 2015; Clawson et al., 2018), we have not previously investigated how these responses change within blocks of a repeated phase-reversing stimulus, as described above using calcium imaging. Given our observation that calcium responses in cell bodies of layer 4 undergo pronounced adaptation across this timescale once a stimulus has become familiar over days, but not when it is novel (Figure 4), we wanted to determine whether a comparable effect is apparent using VEPs. Because the VEP rides on top of ongoing EEG activity and is recorded in awake animals, the evoked potential varies greatly from one phase reversal to the next. This variability is usually overcome by averaging across blocks of many phase reversals. For the analysis included here, we sought to compare response magnitude for the first phase reversal in a block with the last. Overcoming the issue of response variability, therefore, required a large cohort of animals to gain an effective measure of the VEP magnitude on just the first phase reversal of a block or the last. In 34 head-fixed C57BL/6J mice, we recorded VEPs across five blocks of 200 phase reversals (2 Hz) of X° on each of six consecutive days. On a final test day, blocks of the now highly familiar X° orientation were pseudo-randomly interleaved with blocks of a novel X + 90° stimulus, in a protocol that was comparable with that used for the calcium imaging analysis in most regards (Figure 4A), and typical for SRP protocols (Kaplan et al., 2016). By averaging VEP magnitudes across animals for each phase reversal we noted clear changes in VEP magnitudes within each block (Figures 5A,B). In contrast to the effects observed with cell body calcium imaging (Figures 4D,E), we found that adaptation was present when the stimulus was novel but not when the stimulus was familiar. This phenomenon was apparent but not statistically significant on day 1 compared with day 4 (Figure 5C, day 1 first VEP mag = 499.7 ± 22.1 μV; day 1 last, 428.8 ± 28.6 μV; day 4 first, 596.1 ± 33.3 μV; day 4 last, 569.1 ± 37.0 μV; 1-way ANOVA with Tukey’s *post hoc* test for multiple comparisons: Day 1 first and last, *p* = 0.366; Day 4 first and last, *p* = 0.926; First on day 1 and day 4, *p* = 0.124; Last on day 1 and day 4, *p* = 0.008). However, when novel and familiar stimuli were interleaved on the final test day, significant adaptation only occurred to the novel stimulus (Figure 5D, Familiar first VEP mag = 593.6 ± 34.6 μV; Familiar last, 593.8 ± 32.4 μV; Novel first, 601.9 ± 29.4 μV; Novel last, 451.9 ± 22.5 μV; 1-way ANOVA with Tukey’s *post hoc* test for multiple comparisons: familiar first and last, *p* = 1.00; Novel first and last, *p* < 0.003; First for familiar and novel, *p* = 0.997; Last for familiar and novel, *p* = 0.006). Thus, although significant within-block adaptation can be measured with VEPs, this measure again yielded an opposing effect from the somatic calcium imaging in regard to the interaction between short-term adaptation and long-term stimulus familiarity.

### Changes in Neuropil Calcium Responses Resemble Changes in VEPs

**Figure 6:**
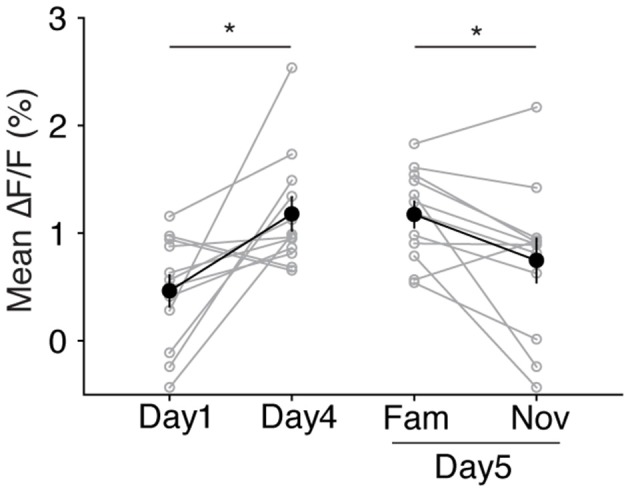
The neuropil response increases to the familiar visual stimulus following SRP induction. Four days of SRP induction increases the neuropil response amplitude to the familiar (X°) grating stimulus, compared to the day 1 response (paired *t*-test, *p* = 0.011). The familiar (X°) visual stimulus on day 5 evokes a higher calcium response in neuropil compared to the novel (X + 90°) visual stimulus (paired *t*-test, *p* = 0.024). Error bars indicate SEM. *p < 0.05.

The observation that changes in layer 4 somatic calcium transients and VEPs were essentially mirror images of one another, as shown in Figures 3–5, was unexpected. VEPs are field potentials representing the sum of ionic currents flowing across the membranes of radially aligned dendrites near the implanted tungsten microelectrode. The negative component of the VEP in mice is maximal at the depth of layer 4, and it has been established through current-source density analysis that increased amplitude of this field potential reflects increased inward (depolarizing) synaptic current (Mitzdorf, 1985; Kirkwood and Bear, 1994; Aizenman et al., 1996). Since endoscopic calcium imaging allows for visualization not only of somatic responses but also of neuropil fluorescence (emanating from *Scnn1a-Cre*-positive cell dendrites), we analyzed the “background” ROIs that were originally selected for correcting somatic responses (see “Materials and Methods” section). Strikingly, this analysis revealed changes that correspond to what is observed in the VEP. The presumptive neuropil response to the familiar (X°) grating stimulus was increased on day 4 of SRP induction compared to the response on day 1 (Figure 6, day 1 ΔF/F = 0.46 ± 0.147%; day 4, 1.18 ± 0.155%; *p* = 0.011 paired *t*-test). Furthermore, the neuropil response to the familiar (X°) stimulus was maintained at this higher magnitude on the test day (day 5), and was significantly higher than the response to the novel (X + 90°) stimulus (Figure 6, day 5 familiar, 1.17 ± 0.121%; novel, 0.75 ± 0.205%; *p* = 0.024 paired *t*-test).

### Orientation Selectivity to Drifting Gratings Is Not Altered by SRP Induction

A characteristic feature of SRP is exquisite selectivity for the features of the stimulus used to induce it (Frenkel et al., 2006; Cooke and Bear, 2010). We were therefore interested to know if induction of SRP with a phase-reversing grating of a single orientation affects the orientation preference of layer 4 neurons assayed with drifting gratings. Our objective was to assay orientation tuning 1 day before (day 0) and 1 day after (day 5) induction of SRP. To mitigate response plasticity caused by exposure of the mice to the drifting gratings, orientation tuning was assessed in mice treated acutely with the NMDA receptor antagonist CPP [3-(2-Carboxypiperazin-4-yl) propyl-1-phosphonic acid; 10 mg/kg i.p.], which blocks the induction of SRP (Frenkel et al., 2006). In a cohort of six mice, we confirmed that prior exposure to CPP did not interfere with the induction of subsequent SRP days later (data not shown).

**Figure 7:**
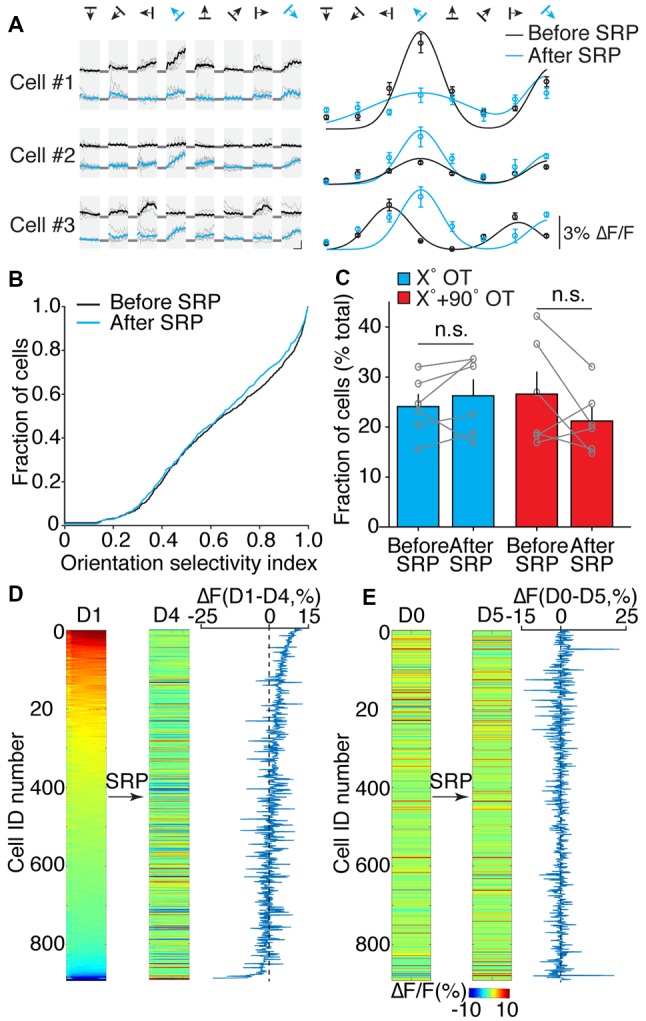
SRP induction with phase-reversal gratings does not alter orientation selectivity of layer 4 neurons to drifting gratings. **(A)** Left: cellular calcium responses from three cells to the eight different directions of a drifting grating visual stimulus before (black) and after SRP induction (blue) and their orientation tuning curves (Right). Black arrows: direction of drifting grating visual stimulus, blue arrows: trained orientation, gray lines: baseline of ΔF/F, circles: the mean magnitude of calcium responses to each direction before (black) and after SRP induction (blue). **(B)** Cumulative distribution of orientation selectivity index (OSI) for all recorded cells (*n* = 891) on day 0 (before SRP, black) and day 5 (after SRP, blue) shows no net change in the proportion of orientation tuned neurons to drifting gratings (2-sample KS test, *p* = 0.129). **(C)** Fraction of cells tuned to the trained (X°) orientation (blue bars) and the fraction of orthogonally (X + 90°) tuned cells (red bars) do not change significantly following the induction of SRP. (Unpaired *t*-test, blue bars, *p* = 0.677; red bars, *p* = 0.344; n.s., not significant). **(D)** Heat map of cellular calcium responses to *phase-reversal* of familiar (X°) orientation visual stimulus from all recorded neurons (*n* = 891) from six animals on day 1 (D1) and day 4 (D4) of SRP induction. Right panel: difference in calcium response amplitude between day 1 and day 4 with the same order of neurons presented at heat map on the left. **(E)** Heat map of cellular calcium responses to *drifting gratings* with the same familiar (X°) orientation visual stimulus with the same order of neurons as presented in part **(D)** on the day before (D0), and after induction of SRP (D5). Right panel: difference in calcium response amplitude between day 0 and day 5 with the same order of neurons presented at heat map on the left.

Orientation tuning was then assessed in 891 neurons in a separate group of six mice. Representative responses to drifting gratings of different orientations and directions are shown in Figure 7A for days 0 and 5, before and after induction of SRP with phase-reversing gratings at 135°. As these examples show, there was considerable variation in how neurons responded when re-tested for orientation preference after the SRP protocol. We first asked if the orientation tuning was altered in the entire population of neurons by calculating for each cell the value of an OSI (see “Materials and Methods” section). As shown in Figure 7B, this analysis revealed no change across the population in the proportion of orientation tuned neurons assayed with drifting gratings. We next investigated whether the fraction of neurons preferring drifting gratings at the familiar orientation (X°) was changed by SRP, and it was not. Similarly, there was no change in the fraction of neurons preferring the novel X + 90° orientation (Figure 7C, X° OT before SRP, 24.07 ± 2.73%; after SRP, 25.96 ± 3.41%; *p* = 0.677 unpaired *t*-test; X + 90° OT before SRP, 27.10 ± 5.19%; after SRP, 21.06 ± 3.02%; *p* = 0.344 unpaired *t*-test). Analysis of neuropil also revealed no change in the response to drifting orientation X° or X + 90° after SRP induced by phase-reversing stimuli. Together, these analyses suggested that response modification elicited by exposure to phase-reversing gratings does not transfer to responses elicited by drifting gratings. Indeed, we found little correlation between the absolute response amplitudes evoked by phase-reversing and drifting stimuli, or on the modification of these responses by the SRP protocol (Figures 7D,E).

## Discussion

In the present study, we characterized the changes in cellular calcium transients in layer 4 of mouse V1 that accompany the induction of SRP, a robust and durable form of cortical response plasticity. Longitudinal two-photon calcium imaging results showed that there was no significant net change across days in the number of active neurons during exposure to the same visual stimulus. However, chronic endoscopic calcium imaging of layer 4 excitatory neurons revealed an overall reduction of cellular responses evoked by phase reversals of the familiar visual stimulus. This reduction in activity emerged across days of training and was accompanied by strong habituation of responses to repetitive stimulation. However, experience-dependent alterations in response to the phase-reversing gratings did not influence orientation tuning to drifting gratings. Taken together, the results suggest that the stimulus selectivity of this type of visual recognition memory includes the spatiotemporal properties of the stimulus and that potentiated short-latency synaptic excitation revealed by the VEP likely recruits within V1 strong shunting inhibition to suppress longer latency cellular responses in layer 4 principal cells.

Had it not been for the history of SRP studies in mouse V1, we might have anticipated that passive viewing of a stimulus that portends neither reward nor punishment would lead over days to reduced evoked activity in primary sensory cortex of awake mice (Kato et al., 2015; Makino and Komiyama, 2015). However, since SRP was first reported over a decade ago (Frenkel et al., 2006), it has been shown in numerous studies that daily exposure of awake head-fixed mice to a phase-reversing grating of a constant orientation reliably *increases* VEP amplitude in layer 4 (reviewed by Cooke and Bear, 2014). The increased VEP amplitude has been shown through current-source density analysis to reflect a potentiated synaptic current sink—an increase in the stimulus-triggered entry of positive charge into dendrites traversing layer 4—and to correlate with an increase in layer 4 multiunit spiking activity at short latencies (Cooke et al., 2015). SRP is extraordinarily selective for the features of the grating stimulus used to elicit it, including orientation, spatial frequency, and contrast. Further, SRP induced by stimulation of one eye does not transfer to responses through the other eye. Although many of these features would be consistent with a subcortical locus of modification, SRP reflects synaptic plasticity within V1 because it is prevented by local treatment in V1 with an NMDA receptor antagonist, a virus to genetically knockdown NMDA receptors (Cooke et al., 2015), and a virus expressing peptides that interfere with activity-dependent delivery of synaptic AMPA receptors (Frenkel et al., 2006). Further, SRP is reversed by local injection of the protein kinase M zeta-pseudosubstrate inhibitory peptide (Cooke and Bear, 2010). Together, these properties of SRP resemble those of the phenomenon of long-term potentiation (LTP), and indeed the induction of LTP by tetanic electrical stimulation of the dorsal lateral geniculate nucleus occludes SRP, and vice versa (Cooke and Bear, 2010). Unlike canonical LTP, however, SRP is not expressed immediately, but emerges over the course of several hours. During this consolidation period, it has been shown that sleep is a critical variable (Aton et al., 2014; Clawson et al., 2018) for subsequent SRP expression. In these respects, SRP has properties expected for perceptual learning—exquisite stimulus selectivity, a requirement for sleep during a consolidation period, and durable expression over long time periods (Fiorentini and Berardi, 1980; Karni and Sagi, 1993; Karni et al., 1994). Thus, a reasonable conclusion was that SRP reflects a modification of thalamocortical excitatory synaptic transmission that may serve perceptual learning (Cooke and Bear, 2010).

This early view of SRP was challenged by a number of subsequent discoveries, however. For example, the different responses to familiar and novel stimuli disappear when monosynaptic thalamocortical VEPs are pharmacologically isolated in layer 4 (Khibnik et al., 2010; Cooke and Bear, 2014). Additionally, local pharmacogenetic silencing of PV+ inhibitory neurons (or blocking NMDA receptors on them with ketamine) acutely interferes with the expression of SRP: VEPs to novel stimuli increase in magnitude, mimicking and occluding the increase observed with familiar stimuli (Kaplan et al., 2016). PV+ neurons mediate fast feed-forward inhibition of layer 4 principal cells, and modulate the amplitude of VEPs and short-latency spiking activity (Swadlow, 2003; Cruikshank et al., 2007; Kaplan et al., 2016; Tremblay et al., 2016). These findings indicate that although feed-forward excitatory synaptic modification may be necessary, it is not sufficient to account for SRP. Furthermore, it also remains to be determined if passive viewing of oriented grating stimuli actually induces perceptual learning, an improvement in an animal’s ability to discriminate orientations. Assayed with drifting gratings, we did not discern a change in the fraction of tuned neurons or a shift in orientation preference in layer 4 as SRP develops. This finding alone does not rule out a contribution of SRP to visual perception in mice, particularly considering the subtlety of cellular changes reported to accompany perceptual learning in monkey V1 (Schoups et al., 2001; Ghose et al., 2002; Moldakarimov et al., 2014; Yan et al., 2014). Nevertheless, a noteworthy difference between the protocols used to elicit SRP and those used to demonstrate perceptual learning is that no behavioral reinforcement is used for SRP.

A robust behavioral correlate of SRP is the long-term habituation of a reflexive movement toward the (now) familiar visual stimulus (Cooke et al., 2015). The cellular changes we observed in the current study appear to parallel these behavioral results, and are consistent with findings in other sensory systems (Kato et al., 2015). Still, the dramatic reversal in the polarity of the changes recorded with cell body calcium transients, vs. VEPs or neuropil calcium transients, was unexpected. VEPs have been used for decades for the assessment of visual detection thresholds (Campbell and Maffei, 1970; Campbell and Kulikowski, 1972; Sherman, 1979; Porciatti et al., 1999; Norcia, 2013), so it was natural to use chronic VEP measurements in mice to study response modification following manipulations of visual experience. In the case of monocular deprivation in mice, for example, reduced VEP amplitude correlates with diminished cortical responsiveness measured by single-unit recordings, imaging of calcium transients and blood oxygenation, and expression of immediate early genes (Smith et al., 2009). However, VEPs in layer 4 are short-latency responses, reflecting net synaptic current flow that is strongly influenced by the relative strength of feedforward excitation and inhibition. In contrast, calcium transients reflect the slower integrated cellular consequences of intracortical synaptic activity. The most straightforward resolution of the conflict between VEP potentiation and calcium response depression in the cell bodies is the recruitment by strengthened feedforward activity of inhibition that clamps cellular output at longer latencies. Indeed, two imaging studies reached a similar conclusion of increased inhibition when mice receive daily passive sensory stimulation (Kato et al., 2015; Makino and Komiyama, 2015). In both of these studies, the increased inhibition was mediated by somatostatin-positive (SOM+) interneurons. Furthermore, in a particularly relevant recent study, it was shown that days of passive exposure to unrewarded stimuli in a virtual reality environment cause a net reduction of activity in layer 2/3 pyramidal neurons and PV+ interneurons, consistent with our findings, and a net increase in the activation of a subset of SOM+ neurons (Khan et al., 2018). In future studies, it will be of great interest to test the hypothesis that a specific population of inhibitory neurons is responsible for the response depression we observe in layer 4 principal cells following induction of SRP.

Electrophysiological recordings of action potentials after SRP have shown increased peak firing rate for familiar stimuli over novel stimuli in layer 4 (Cooke et al., 2015) and in infragranular layers (Aton et al., 2014; Clawson et al., 2018). Although these findings may seem to be at odds with our calcium imaging results, the two sets of results may be reconciled based on the hypothesis that increased clamping inhibition arises from increased familiarity. The transient increase in multi-unit firing of neurons in thalamo-recipient layer 4 as a result of SRP occurs at very short latencies, before the strong recruitment of polysynaptic inhibition. Our calcium imaging results, therefore, support a clear prediction that while unit firing may be briefly elevated at short latencies after familiar stimulus phase reversal, it should be suppressed relative to a novel stimulus at longer latencies. Future studies will be undertaken to test this prediction in which action potentials are recorded with electrodes while imaging GCaMP signal from the same neurons. Imaging the neuropil captures calcium transients within postsynaptic dendrites, which are expected to more closely correspond to the summed synaptic currents measured with VEPs (Mitzdorf, 1985). Our findings show that the neuropil GCaMP signal does indeed very faithfully adhere to the changes documented with VEPs across days and within sessions. Some of the VEP changes, therefore, are likely to reflect modification of net synaptic excitation in the dendrites of layer 4 neurons. This finding presents an opportunity to undertake higher resolution imaging that targets individual synapses to gain a deeper understanding of the circuit modifications underlying SRP, particularly if allied with genetic strategies to isolate particular cell types, as we have here using the *Scnn1a-Cre* recombinase line.

Habituation to stimuli that portend neither reward nor punishment is vitally important for the allocation of neural resources to the detection of novelty or other potentially important environmental features. A failure to habituate to innocuous sensory stimuli is a common symptom of neuropsychiatric diseases characterized by impairments in cognition (e.g., see Sinha et al., 2014). Thus, understanding the underlying synaptic, cellular and circuit mechanisms of long-term habituation to sensory stimuli offers the opportunity to reveal the pathophysiology of these diseases, and perhaps provide guidance toward novel therapeutic approaches.

## Data Availability Statement

The datasets generated for this study are available on request to the corresponding author.

## Ethics Statement

The animal study was reviewed and approved by the Committee on Animal Care at Massachusetts Institute of Technology. All experiments were performed in accordance with the guidelines of the National Institutes of Health.

## Author Contributions

TK conducted all calcium imaging experiments and VEP recordings in GCaMP expressing mice and conducted all analyses on the resulting data in Figures 1–4 and 6, 7. MH contributed to the analysis of imaging data and experimental design. FC and SC acquired, analyzed and interpreted all VEP data in Figure 5. TK and MB participated in the design of all experiments, interpreted results and wrote the manuscript.

## Conflict of Interest

The authors declare that the research was conducted in the absence of any commercial or financial relationships that could be construed as a potential conflict of interest.
